# Diagnostic Performance of Artificial Intelligence-Based Computer-Aided Detection and Diagnosis in Pediatric Radiology: A Systematic Review

**DOI:** 10.3390/children10030525

**Published:** 2023-03-08

**Authors:** Curtise K. C. Ng

**Affiliations:** 1 Curtin Medical School, Curtin University, GPO Box U1987, Perth, WA 6845, Australia; curtise.ng@curtin.edu.au or curtise_ng@yahoo.com.hk; Tel.: +61-8-9266-7314; Fax: +61-8-9266-2377; 2 Curtin Health Innovation Research Institute (CHIRI), Faculty of Health Sciences, Curtin University, GPO Box U1987, Perth, WA 6845, Australia

**Keywords:** children, confusion matrix, convolutional neural network, deep learning, diagnostic accuracy, disease identification, image interpretation, machine learning, medical imaging, pneumonia

## Abstract

Artificial intelligence (AI)-based computer-aided detection and diagnosis (CAD) is an important research area in radiology. However, only two narrative reviews about general uses of AI in pediatric radiology and AI-based CAD in pediatric chest imaging have been published yet. The purpose of this systematic review is to investigate the AI-based CAD applications in pediatric radiology, their diagnostic performances and methods for their performance evaluation. A literature search with the use of electronic databases was conducted on 11 January 2023. Twenty-three articles that met the selection criteria were included. This review shows that the AI-based CAD could be applied in pediatric brain, respiratory, musculoskeletal, urologic and cardiac imaging, and especially for pneumonia detection. Most of the studies (93.3%, 14/15; 77.8%, 14/18; 73.3%, 11/15; 80.0%, 8/10; 66.6%, 2/3; 84.2%, 16/19; 80.0%, 8/10) reported model performances of at least 0.83 (area under receiver operating characteristic curve), 0.84 (sensitivity), 0.80 (specificity), 0.89 (positive predictive value), 0.63 (negative predictive value), 0.87 (accuracy), and 0.82 (F1 score), respectively. However, a range of methodological weaknesses (especially a lack of model external validation) are found in the included studies. In the future, more AI-based CAD studies in pediatric radiology with robust methodology should be conducted for convincing clinical centers to adopt CAD and realizing its benefits in a wider context.

## 1. Introduction

Artificial intelligence (AI) is an active research area in radiology [1,2,3,4]. However, the investigation of use of AI for computer-aided detection and diagnosis (CAD) in radiology started in 1955. Any CAD systems are AI applications and can be subdivided into two types: computer-aided detection (CADe) and computer-aided diagnosis (CADx) [5,6,7]. The former focuses on the automatic detection of anomalies (e.g., tumor, etc.) on medical images, while the latter is capable of automatically characterizing anomaly types such as benign and malignant [7]. Since the 1980s, more researchers have become interested in the CAD system development due to availabilities of digital medical imaging and powerful computers. The first CAD system approved by The United States of America Food and Drug Administration was commercially available in 1998 for breast cancer detection [6].

Early AI-based CAD systems in radiology were entirely rule based, and their algorithms could not improve automatically. In contrast, machine learning (ML)-based and deep learning (DL)-based CAD systems can automatically improve their performances through training, and hence, they have become dominant. DL is a subset of ML, and its models have more layers than those of ML. The DL algorithms are capable of modeling high-level abstractions in medical images without predetermined inputs [5,8,9].

A recent systematic review has shown that the DL-based CAD systems in radiology have been developed for a range of areas including breast, cardiovascular, gastrointestinal, hepatological, neurological, respiratory, rheumatic, thyroid and urologic diseases, and trauma. The performances of these CAD systems matched expert readers’ capabilities (pooled sensitivity and specificity: 87.0% vs. 86.4% and 92.5% vs. 90.5%), respectively [10]. Apparently, the current AI-based CAD systems might help to address radiologist shortage problems [9,10,11]. Nevertheless, various systematic reviews have criticized that the diagnostic performance figures reported in many AI-based CAD studies were not trustworthy because of their methodological weaknesses [10,12,13].

Pediatric radiology is a subset of radiology [14,15,16,17]. The aforementioned systematic review findings may not be applicable to the pediatric radiology [10,12,13,16,17]. For example, the AI-based CAD systems for breast and prostate cancer detections seem not relevant to children [10,12,13,17]. Although the AI-based CAD is an important topic area in radiology [10,12,13], apparently, only two narrative reviews about various uses of AI in pediatric radiology (e.g., examination booking, image acquisition and post-processing, CAD, etc.) [17] and AI-based CAD in pediatric chest imaging have been published to date [16]. Hence, it is timely to conduct a systematic review about the diagnostic performance of AI-based CAD in pediatric radiology. The purpose of this article is to systematically review the original studies to answer the question: “What are the AI-based CAD applications in pediatric radiology, their diagnostic performances and methods for their performance evaluation?”

## 2. Materials and Methods

This systematic review of the diagnostic performance of the AI-based CAD in pediatric radiology was conducted as per the preferred reporting items for systematic reviews and meta-analyses (PRISMA) guidelines and patient/population, intervention, comparison, and outcome model. This involved a literature search, article selection, and data extraction and synthesis [10,12,13,14,18].

### 2.1. Literature Search

The literature search with the use of electronic scholarly publication databases, including EBSCOhost/Cumulative Index of Nursing and Allied Health Literature Ultimate, Ovid/Embase, PubMed/Medline, ScienceDirect, Scopus, SpringerLink, Web of Science, and Wiley Online Library was conducted on 11 January 2023 to identify articles investigating the diagnostic performance of the AI-based CAD in the pediatric radiology with no publication year restriction [12,19,20]. The search statement used was (“Artificial Intelligence” OR “Machine Learning” OR “Deep Learning”) AND (“Computer-Aided Diagnosis” OR “Computer-Aided Detection”) AND (“Pediatric” OR “Children”) AND (“Radiology” OR “Medical Imaging”). The keywords used in the search were based on the review focus and systematic reviews on the diagnostic performance of the AI-based CAD in radiology [19,20,21,22,23].

### 2.2. Article Selection

A reviewer with more than 20 years of experience in conducting literature reviews was involved in the article selection process [14,24]. Only peer-reviewed original research articles that were written in English and focused on the AI-based CAD in pediatric radiology with the diagnostic accuracy measures were included. Gray literature, conference proceedings, editorials, review, perspective, opinion, commentary, and non-peer-reviewed (e.g., those published via the arXiv research-sharing platform, etc.) articles were excluded because this systematic review focused on the diagnostic performance of the AI-based CAD in the pediatric radiology and appraisal of the associated methodology reported in the refereed original articles. Papers mainly about image segmentation or clinical prediction instead of disease identification or classification were also excluded [12].

**Figure 1:**
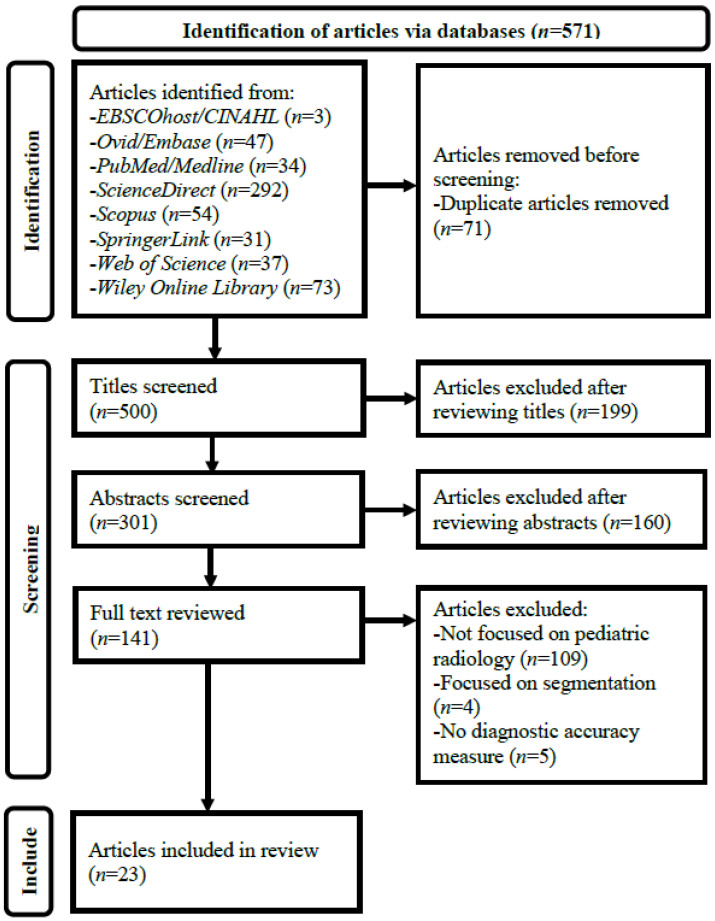
Preferred reporting items for systematic reviews and meta-analyses flow diagram for systematic review of diagnostic performance of artificial intelligence-based computer-aided detection and diagnosis in pediatric radiology. CINAHL, Cumulative Index of Nursing and Allied Health Literature.

Figure 1 illustrates the details of the article selection process. A three-stage screening process through assessing (1) article titles, (2) abstracts, and (3) full texts against the selection criteria was employed after duplicate article removal from the results of the database search. Every non-duplicate article within the search results was retained until its exclusion could be decided [14,25,26].

### 2.3. Data Extraction and Synthesis

Two data extraction forms (Table 1 and Table 2) were developed based on a recent systematic review on the diagnostic performance of AI-based CAD in radiology [12]. The data, including author name and country, publication year, imaging modality, diagnosis, diagnostic performance of AI-based CAD system (area under receiver operating characteristic curve (AUC), sensitivity, specificity, positive predictive value (PPV), negative predictive value (NPV), accuracy and F1 score), AI type (such as ML and DL) and model (e.g., support vector machine, convolutional neural network (CNN), etc.) for developing the CAD system, study design (either prospective or retrospective), source (such as public dataset by Guangzhou Women and Children’s Medical Center, China) and size (e.g., 5858 images, etc.) of dataset for testing the CAD system, patient/population (such as 1–5-year-old children), any sample size calculation, model internal validation type (e.g., 10-fold cross-validation, etc.), any model external validation (i.e., any model testing with use of dataset not involved in internal validation and acquired from different setting), reference standard for ground truth establishment (such as histology and expert consensus), any model performance comparison with clinician and model commercial availability were extracted from each included paper. When diagnostic performance findings were reported for multiple AI-based CAD models in a study, only the values of the best performing model were presented [27]. Meta-analysis was not conducted because this systematic review covered a range of imaging modalities and pathologies, and hence, high study heterogeneity was expected, affecting its usefulness [12,13,28]. The Revised Quality Assessment of Diagnostic Accuracy Studies (QUADAS-2) tool was used to assess the quality of all included studies [9,12,13,19,23,27,29].

## 3. Results

Twenty-three articles met the selection criteria and were included in this review [30,31,32,33,34,35,36,37,38,39,40,41,42,43,44,45,46,47,48,49,50,51,52]. Table 1 shows their AI-based CAD application areas in the pediatric radiology and the diagnostic performances. These studies covered brain (*n* = 9) [30,31,32,33,34,35,36,37,38], respiratory (*n* = 9) [42,43,44,45,46,47,48,49,50], musculoskeletal (*n* = 2) [40,41], urologic (*n* = 2) [51,52] and cardiac imaging (*n* = 1) [39]. The commonest AI-based CAD application area (30.4%, 7/23) was pediatric pneumonia [43,45,46,47,48,49,50]. No study reported all seven diagnostic accuracy measures [30,31,32,33,34,35,36,37,38,39,40,41,42,43,44,45,46,47,48,49,50,51,52]. Most commonly, the papers (30.4%, 7/23) reported four metrics [30,32,35,42,44,45,52]. Accuracy (*n* = 19) and sensitivity (*n* = 18) were the two most frequently used evaluation metrics [30,31,32,33,34,35,36,37,38,39,41,42,43,44,45,46,47,48,49,50,51,52]. One study only used one measure, AUC [40]. Most of the articles (93.3%, 14/15; 77.8%, 14/18; 73.3%, 11/15; 80.0%, 8/10; 66.6%, 2/3; 84.2%, 16/19; 80.0%, 8/10) reported AI-based CAD model performances of at least 0.83 (AUC), 0.84 (sensitivity), 0.80 (specificity), 0.89 (PPV), 0.63 (NPV), 0.87 (accuracy), and 0.82 (F1 score), respectively. The ranges of the reported performance values were 0.698–0.999 (AUC), 0.420–0.987 (sensitivity), 0.585–1.000 (specificity), 0.600–1.000 (PPV), 0.260–0.971 (NPV), 0.643–0.986 (accuracy), and 0.626–0.983 (F1 score) [30,31,32,33,34,35,36,37,38,39,40,41,42,43,44,45,46,47,48,49,50,51,52]. For the seven studies about AI-based CAD for pneumonia, their model performances were at least 0.850 (AUC), 0.760 (sensitivity), 0.800 (specificity), 0.891 (PPV), 0.905 (accuracy) and 0.903 (F1 score).

**Table 1:** Artificial intelligence-based computer-aided detection and diagnosis application areas in pediatric radiology and their diagnostic performances.

Author, Year and Country	Modality	Diagnosis	Diagnostic Performance						
			AUC	Sensitivity	Specificity	PPV	NPV	Accuracy	F1 Score
Brain Imaging									
Dou et al. (2022)—China [30]	MRI	Bipolar disorder	0.830	0.909	0.769	NR	NR	0.854	NR
Kuttala et al. (2022)—Australia, India & United Arab Emirates [31]	MRI	ADHD and ASD	0.850 (ADHA); 0.910 (ASD)	NR	NR	NR	NR	0.854 (ADHA); 0.978 (ASD)	NR
Li et al. (2020)—China [32]	MRI	Posterior fossa tumors	0.865	0.929	0.800	NR	NR	0.878	NR
Peruzzo et al. (2016)—Italy [33]	MRI	Malformations of corpus callosum	0.953	0.923	0.904	0.906	NR	0.914	NR
Prince et al. (2020)—USA [34]	CT & MRI	ACP	0.978	NR	NR	NR	NR	0.979	NR
Tan et al. (2013)—USA [35]	MRI	Congenital sensori-neural hearing loss	0.900	0.890	0.860	NR	NR	0.870	NR
Xiao et al. (2019)—China [36]	MRI	ASD	NR	0.980	0.936	0.959	0.971	0.963	NR
Zahia et al. (2020)—Spain [37]	MRI	Dyslexia	NR	0.750	0.714	0.600	NR	0.727	0.670
Zhou et al. (2021)—China [38]	MRI	ADHD	0.698	0.609	0.676	NR	NR	0.643	0.626
Cardiac Imaging									
Lee et al. (2022)—South Korea [39]	US	Kawasaki disease	NR	0.841	0.585	0.811	0.633	0.759	0.826
Musculoskeletal Imaging									
Petibon et al. (2021)—Canada, Israel and USA [40]	SPECT	Low back pain	0.830	NR	NR	NR	NR	NR	NR
Sezer and Sezer (2020)—France and Turkey [41]	US	DDH	NR	0.962	0.980	NR	NR	0.977	NR
Respiratory Imaging									
Behzadi—Khormouji et al. (2020)—Iran and USA [42]	X-ray	Pulmonary consolidation	0.995	0.987	0.864	NR	NR	0.945	NR
Bodapati and Rohith (2022)—India [43]	X-ray	Pneumonia	0.939	NR	NR	NR	NR	0.948	0.959
Helm et al. (2009)—Canada, UK and USA [44]	CT	Pulmonary nodules	NR	0.420	1.000	1.000	0.260	NR	NR
Jiang and Chen (2022)-China [45]	X-ray	Pneumonia	NR	0.894	NR	0.918	NR	0.912	0.903
Liang and Zheng (2020)-China [46]	X-ray	Pneumonia	0.953	0.967	NR	0.891	NR	0.905	0.927
Mahomed et al. (2020)-Netherlands and South Africa [47]	X-ray	Primary-endpoint pneumonia	0.850	0.760	0.800	NR	NR	NR	NR
Shouman et al. (2022)-Egypt and Saudi Arabia [48]	X-ray	Bacterial and viral pneumonia	0.999	0.987	0.987	0.979	NR	0.986	0.983
Silva et al. (2022)-Brazil [49]	X-ray	Pneumonia	NR	0.945	NR	0.957	NR	NR	0.951
Vrbančič and Podgorelec (2022)-Slovenia [50]	X-ray	Pneumonia	0.952	0.976	0.927	0.973	NR	0.963	0.974
Urologic Imaging									
Guan et al. (2022)-China [51]	US	Hydronephrosis	NR	NR	NR	NR	NR	0.891	0.895
Zheng et al. (2019)-China and USA [52]	US	CAKUT	0.920	0.86	0.880	NR	NR	0.870	NR

ACP, adamantinomatous craniopharyngioma; ADHD, attention deficit hyperactivity disorder; ASD, autism spectrum disorder; AUC, area under receiver operating characteristic curve; CAKUT, congenital abnormalities of kidney and urinary tract; CT, computed tomography; DDH, developmental dysplasia of hip; MRI, magnetic resonance imaging; NPV, negative predictive value; NR, not reported; PPV, positive predictive value; SPECT, single-photon emission computed tomography; UK, United Kingdom; US, ultrasound; USA, United States of America.

Table 2 presents the included study characteristics. Overall, 18 out of 23 (78.3%) studies were published in the last three years [30,31,32,34,37,38,39,40,41,42,43,45,46,47,48,49,50,51]. Most of them (72.7%, 16/22) developed the DL-based CAD systems [31,34,36,37,39,40,41,42,43,45,46,48,49,50,51,52]. Of these 16 DL-based systems, 75% (*n* = 12) used the CNN model [34,37,39,40,41,42,43,46,48,49,50,51]. Magnetic resonance imaging (MRI) (*n* = 9) [30,31,32,33,34,35,36,37,38] and X-ray (*n* = 8) [42,43,45,46,47,48,49,50] were most frequently used by the AI-based CAD models for the brain and respiratory disease diagnoses, respectively. The majority of studies (69.6%, 16/23) collected the datasets retrospectively [31,33,34,36,38,39,40,42,43,44,45,46,48,49,50,52]. Of these 16 retrospective studies, about one-third (*n* = 11) relied on the public datasets [31,34,36,38,42,43,45,46,48,49,50]; most of them (*n* = 7) used the chest X-ray dataset consisting of 1741 normal and 4346 pneumonia images of 6087 1–5-year-old children collected from the Guangzhou Women and Children’s Medical Center, China [42,43,45,46,48,49,50]. No study calculated the sample size for the data collection [30,31,32,33,34,35,36,37,38,39,40,41,42,43,44,45,46,47,48,49,50,51,52]. Most of the studies (60.9%, 14/23) collected less than 233 cases [30,31,32,33,34,35,36,37,38,39,40,41,44,52], and about one-third (*n* = 7) collected data of less than 87 patients for testing their systems [30,32,34,35,37,40,44]. Hence, for the model internal validation, more than half of the studies (*n* = 13) used the cross-validation to address the small test set issue [30,33,34,35,36,37,38,39,40,47,50,51,52]. However, all but one did not conduct the external validation [30,31,32,33,34,35,36,37,38,39,40,41,42,43,45,46,47,48,49,50,51,52]. The only exception conducted external validation for a commercial AI-based CAD system evaluation [44]. Less than one-fifth of the included studies (*n* = 4) used the consensus diagnosis as the reference standard (ground truth) for the model training and performance evaluation [33,42,44,47], and one-quarter (*n* = 6) did not report the reference standard [31,43,45,46,48,49]. Only about one-fifth (*n* = 5) compared their model performances with those of clinicians [33,34,40,44,47], and most of these (60%, 3/5) were the studies using the consensus diagnosis as the reference standard [33,44,47].

**Figure 2:**
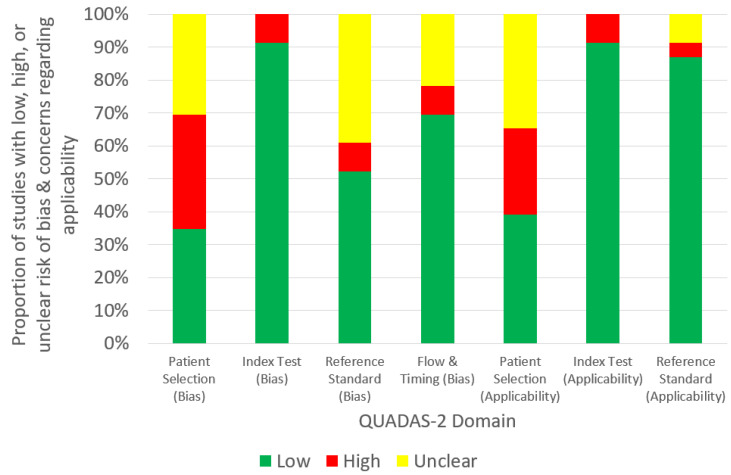
Quality assessment summary of all (23) included studies based on Revised Quality Assessment of Diagnostic Accuracy Studies tool.

Figure 2 shows the quality assessment summary of all (23) studies based on the QUADAS-2 tool. Only around one-third of the studies had a low risk of bias [34,35,36,37,38,41,44,52] and concern regarding applicability for the patient selection category [30,34,35,36,37,38,41,44,52]. The low risk of bias of the reference standard was only noted in about half of them [32,33,34,35,36,37,38,40,42,47,50,52].

**Table 2:** Study characteristics of artificial intelligence-based computer-aided detection and diagnosis in pediatric radiology.

Author, Year and Country	Modality	Diagnosis	AI Type and Model	Study Design	Dataset Source	Test Set Size	Patient/Population	Sample Size Calculation	Internal Validation Type	External Validation	Reference Standard	AI vs. Clinician	Commercial Availability
Brain Imaging													
Dou et al. (2022)—China [30]	MRI	Bipolar disorder	ML-LR	Prospective	Private dataset by Second Xiangya Hospital, China	52 scans	12–18-year-old children	No	2-fold cross-validation	No	Clinical diagnosis	No	No
Kuttala et al. (2022)—Australia, India and United Arab Emirates [31]	MRI	ADHD and ASD	DL-GAN and softmax	Retrospective	Public datasets (ADHD-200 and Autism Brain Imaging Data Exchange II)	217 scans	Children (median ages for baseline and follow-up scans: 12 and 15 years, respectively)	No	NR	No	NR	No	No
Li et al. (2020)—China [32]	MRI	Posterior fossa tumors	ML-SVM	Prospective	Private dataset by Affiliated Hospital of Zhengzhou University, China	45 scans	0–14-year-old children	No	Repeated hold-out with 70:30 random split	No	Histology	No	No
Peruzzo et al. (2016)—Italy [33]	MRI	Malformations of corpus callosum	ML-SVM	Retrospective	Private dataset by Scientific Institute “Eugenio Medea”, Italy	104 scans	2–12-year-old children	No	Leave-one-out cross validation	No	Expert consensus	Yes	No
Prince et al. (2020)—USA [34]	CT and MRI	ACP	DL-CNN	Retrospective	Public dataset (ATPC Consortium) and private datasets by Children’s Hospital Colorado and St. Jude Children’s Research Hospital, USA	86 CT-MRI scans	Children	No	60:40 random split and 5-fold cross validation	No	Histology	Yes	No
Tan et al. (2013)—USA [35]	MRI	Congenital sensori-neural hearing loss	ML-SVM	Prospective	Private dataset by Cincinnati Children’s Hospital Medical Center, USA	39 scans	8–24-month-old children	No	Leave-one-out cross-validation	No	Follow-up	No	No
Xiao et al. (2019)—China [36]	MRI	ASD	DL-SAE and softmax	Retrospective	Public dataset (Autism Brain Imaging Data Exchange II)	198 scans	5–12-year-old children	No	11-, 33-, 66-, 99- and 198-fold cross-validation	No	Clinical diagnosis	No	No
Zahia et al. (2020)—Spain [37]	MRI	Dyslexia	DL-CNN	Prospective	Private dataset by University Hospital of Cruces, Spain	55 scans	9–12-year-old children	No	4-fold cross validation	No	Clinical diagnosis	No	No
Zhou et al. (2021)—China [38]	MRI	ADHD	ML-SVM	Retrospective	Public dataset (Adolescent Brain Cognitive Development Data Repository)	232 scans	9–10-year-old children	No	10-fold cross-validation	No	Clinical diagnosis	No	No
Cardiac Imaging													
Lee et al. (2022)—South Korea [39]	US	Kawasaki disease	DL-CNN	Retrospective	Private dataset by Yonsei University Gangnam Severance Hospital, South Korea	203 scans	Children	No	10-fold cross-validation	No	Single expert reader	No	No
Musculoskeletal Imaging													
Petibon et al. (2021)—Canada, Israel and USA [40]	SPECT	Low back pain	DL-CNN	Retrospective	Private dataset by Boston Children’s Hospital, USA	65 scans	10–17 years old children	No	3-fold cross-validation	No	Other-ground truth established by artificial lesion insertion	Yes	No
Sezer and Sezer (2020)—France and Turkey [41]	US	DDH	DL-CNN	Prospective	Private dataset	203 scans	0–6-month-old children	No	70:30 random split	No	Single expert reader	No	No
Respiratory Imaging													
Behzadi—Khormouji et al. (2020)—Iran and USA [42]	X-ray	Pulmonary consolidation	DL-CNN	Retrospective	Public dataset by Guangzhou Women and Children’s Medical Center, China	582 images	1–5-year-old children	No	90:10 random split	No	Expert consensus	No	No
Bodapati and Rohith (2022)—India [43]	X-ray	Pneumonia	DL-CNN and CapsNet	Retrospective	Public dataset by Guangzhou Women and Children’s Medical Center, China	640 images	1–5-year-old children	No	NR	No	NR	No	No
Helm et al. (2009)—Canada, UK and USA [44]	CT	Pulmonary nodules	NR	Retrospective	Private dataset by a tertiary pediatric hospital	29 scans	3 years and 11 months to 18-year-old children	No	NR	Yes	Expert and reader consensus	Yes	Yes
Jiang and Chen (2022)—China [45]	X-ray	Pneumonia	DL-ViT	Retrospective	Public dataset by Guangzhou Women and Children’s Medical Center, China	624 images	1–5-year-old children	No	NR	No	NR	No	No
Liang and Zheng (2020)—China [46]	X-ray	Pneumonia	DL-CNN	Retrospective	Public dataset by Guangzhou Women and Children’s Medical Center, China	624 images	1–5-year-old children	No	90:10 random split	No	NR	No	No
Mahomed et al. (2020)—Netherlands and South Africa [47]	X-ray	Primary-endpoint pneumonia	ML-SVM	Prospective	Private dataset by Chris Hani Baragwanath Academic Hospital, South Africa	858 digitized images	1–59-month-old children	No	10-fold cross-validation	No	Reader consensus	Yes	No
Shouman et al. (2022)—Egypt and Saudi Arabia [48]	X-ray	Bacterial and viral pneumonia	DL-CNN and LSTM	Retrospective	Public dataset by Guangzhou Women and Children’s Medical Center, China	586 images	1–5-year-old children	No	90:10 random split	No	NR	No	No
Silva et al. (2022)—Brazil [49]	X-ray	Pneumonia	DL-CNN	Retrospective	Public dataset by Guangzhou Women and Children’s Medical Center, China	1172 images	1–5-year-old children	No	NR	No	NR	No	No
Vrbančič and Podgorelec (2022)—Slovenia [50]	X-ray	Pneumonia	DL-CNN and SGD	Retrospective	Public dataset by Guangzhou Women and Children’s Medical Center, China	5858 images	1–5-year-old children	No	10-fold cross-validation	No	Expert readers	No	No
Urologic Imaging													
Guan et al. (2022)—China [51]	US	Hydronephrosis	DL-CNN	Prospective	Private dataset by Beijing Children’s Hospital, China	3257 images	Children	No	10-fold cross-validation	No	Readers and experts without consensus	No	No
Zheng et al. (2019)—China and USA [52]	US	CAKUT	DL-SVM	Retrospective	Private dataset by Children’s Hospital of Philadelphia, USA	100 scans	Children with mean age of 111 days (SD: 262)	No	10-fold cross-validation	No	Clinical diagnosis	No	No

ACP, adamantinomatous craniopharyngioma; ADHD, attention deficit hyperactivity disorder; AI, artificial intelligence; ASD, autism spectrum disorder; ATPC, Advancing Treatment for Pediatric Craniopharyngioma; CAKUT, congenital abnormalities of kidney and urinary tract; CapsNet, capsule network; CNN, convolutional neural network; CT, computed tomography; DDH, developmental dysplasia of hip; DL, deep learning; GAN, generative adversarial network; LR, logistic regression; LSTM, long short-term memory; ML, machine learning; MRI, magnetic resonance imaging; NR, not reported; SAE, stacked auto-encoder; SD, standard deviation; SGD, stochastic gradient descent; SPECT, single-photon emission computed tomography; SVM, support vector machine; UK, United Kingdom; US, ultrasound; USA, United States of America; ViT, vision transformer.

## 4. Discussion

This article is the first systematic review on the diagnostic performance of the AI-based CAD in the pediatric radiology covering the brain [30,31,32,33,34,35,36,37,38], respiratory [42,43,44,45,46,47,48,49,50], musculoskeletal [40,41], urologic [51,52] and cardiac imaging [39]. Hence, it advances the previous two narrative reviews about various uses of AI in the pediatric radiology [17] and the AI-based CAD in the pediatric chest imaging [16] published in 2021 and 2022, respectively. Most of the included studies reported AI-based CAD model performances of at least 0.83 (AUC), 0.84 (sensitivity), 0.80 (specificity), 0.89 (PPV), 0.63 (NPV), 0.87 (accuracy), and 0.82 (F1 score) [30,31,32,33,34,35,36,37,38,39,40,41,42,43,44,45,46,47,48,49,50,51,52]. However, the diagnostic performances of these CAD systems appeared a bit lower than those reported in the systematic review of the AI-based CAD in the radiology (pooled sensitivity and specificity: 0.87 and 0.93, respectively) [10]. In addition, the pediatric pneumonia was the only disease that was investigated by more than two studies [43,45,46,47,48,49,50]. Although these studies reported that their CAD performances for the pneumonia diagnosis were at least 0.850 (AUC), 0.760 (sensitivity), 0.800 (specificity), 0.891 (PPV), 0.905 (accuracy) and 0.903 (F1 score), which would be sufficient to support less experienced pediatric radiologists in image interpretation, all but one were the retrospective studies and relied on the chest X-ray dataset consisting of 1741 normal and 4346 pneumonia images of 6087 1–5-year-old children collected from the Guangzhou Women and Children’s Medical Center, China [13,43,44,45,46,47,48,49,50]. It is noted that the use of the public dataset could facilitate AI-based CAD model performance comparison with other similar studies [43]. On the other hand, this approach would affect the model generalization ability (i.e., unable to maintain the performance when applying to different settings), causing the model to be unfit for real clinical situations [10,46]. Although techniques such as the cross-validation can be used to improve the AI-based CAD model generalization ability [37], only one of these studies used the cross-validation approach [50], while half of them did not report the internal validation type [43,45,49]. In addition, some ground truths given in the public datasets might be inaccurate, indicating potential reference standard issues [10,42]. These studies did not calculate the required sample size; perform the external validation; and compare their model performances with radiologists, but they are essential for the demonstration of the trustworthiness of study findings [43,45,46,48,49,50]. As per Table 2, the aforementioned methodological issues were also common for other included studies. These issues are found in many studies about the AI-based CAD in the radiology as well [10,12,13]. 

Table 2 reveals that the DL and its model, CNN, were commonly used for the development of the AI-based CAD systems in the pediatric radiology similar to the situation in the radiology [13]. According to the recent narrative review about the AI-based CAD in the pediatric chest imaging published in 2022, 144 Conformité Européenne-marked AI-based CAD systems for brain (35%), respiratory, (27%), musculoskeletal (11%), breast (11%), other (7%), abdominal (6%) and cardiac (4%) imaging were commercially available in the radiology [16]. The proportions of these systems are comparable to the findings of this systematic review that the brain, respiratory and musculoskeletal imaging were the three most popular application areas of the AI-based CAD in the pediatric radiology and the cardiac imaging was the least (Table 1). However, except for Helm et al.’s retrospective study about the detection of pediatric pulmonary nodules in 29 3–18-year-old patients with the use of the AI-based CAD system developed for adults [44], no commercial system was involved in the included studies (Table 2) [30,31,32,33,34,35,36,37,38,39,40,41,42,43,45,46,47,48,49,50,51,52]. Helm et al.’s study [44] was the only one that performed the external validation of the CAD system with the reference standard established by the consensus of six radiologists, and one of the few compared the CAD performance with the clinicians. However, that study only used four evaluation measures: sensitivity (0.42), specificity (1.00), PPV (1.00) and NPV (0.26), and the other metrics commonly used in more clinically focused studies, AUC and accuracy, were not reported [10,12,44,53]. This highlights that even for a more clinically focused AI-based CAD study in the pediatric radiology with the better design, the common methodological weaknesses such as the retrospective data collection with limited information of patient characteristics reported and cases included, and no sample size calculation, were still prevalent (Table 2) [44,54,55]. Hence, these explain the findings in Figure 2 that the concern regarding applicability was found in the patient selection, and the risk of bias was noted in both patient selection and reference standard categories, although similar results were also reported in the systematic reviews of the AI-based CAD in the radiology [10,12].

Apparently, the AI-based CAD in the pediatric radiology is less developed when compared to its adult counterpart. For example, not many studies were published before 2020 [33,35,36,44,52,56,57,58,59,60,61,62,63,64,65,66,67,68,69,70,71,72,73,74,75,76], and the studies mainly focused on the MRI and X-ray and particular patient cohorts [30,31,32,33,34,35,36,37,38,39,40,41,42,43,44,45,46,47,48,49,50,51,52] (Table 2). Although Schalekamp et al.’s [16] narrative review published in 2022 suggested the use of the AI-based CAD designed for the adult population in children, Helm et al.’s [44] study demonstrated that this approach yielded low sensitivity (0.42) and NPV (0.26) in detecting pediatric pulmonary nodules because of the smaller nodule sizes in children. Hence, AI-based CAD systems specifically designed/finetuned for the pediatric radiology by researchers and/or commercial companies seem necessary in the future. In addition, for further research, more robust study designs that can address the aforementioned methodological issues (especially the lack of the external validation) are essential for providing trustworthy findings to convince clinical centers to adopt the AI-based CAD in the pediatric radiology. In this way, the potential benefits of the CAD could be realized in a wider context [5,10,12,13]. 

This systematic review has two major limitations. The article selection, data extraction, and synthesis were performed by a single author, albeit one with more than 20 years of experience in conducting the literature reviews [14]. According to a recent methodological systematic review, this is an appropriate arrangement provided that the single reviewer is experienced [14,24,77,78,79]. Additionally, through adherence to the PRISMA guidelines and the use of the data extraction forms (Table 1 and Table 2) devised based on the recent systematic review on the diagnostic performance of the AI-based CAD in the radiology and the QUADAS-2 tool, the potential bias should be addressed to a certain extent [12,14,26,29]. In addition, only articles in English identified via databases were included, potentially affecting the comprehensiveness of this systematic review [9,21,26,27,80]. Nevertheless, this review still has a wider coverage about the AI-based CAD in the pediatric radiology than the previous two narrative reviews [16,17].

## 5. Conclusions

This systematic review shows that the AI-based CAD for the pediatric radiology could be applied in the brain, respiratory, musculoskeletal, urologic and cardiac imaging. Most of the studies (93.3%, 14/15; 77.8%, 14/18; 73.3%, 11/15; 80.0%, 8/10; 66.6%, 2/3; 84.2%, 16/19; 80.0%, 8/10) reported AI-based CAD model performances of at least 0.83 (AUC), 0.84 (sensitivity), 0.80 (specificity), 0.89 (PPV), 0.63 (NPV), 0.87 (accuracy), and 0.82 (F1 score), respectively. The pediatric pneumonia was the most common pathology covered in the included studies. They reported that their CAD performances for pneumonia diagnosis were at least 0.850 (AUC), 0.760 (sensitivity), 0.800 (specificity), 0.891 (PPV), 0.905 (accuracy) and 0.903 (F1 score). Although these diagnostic performances appear sufficient to support the less experienced pediatric radiologists in the image interpretation, a range of methodological weaknesses such as the retrospective data collection, no sample size calculation, overreliance on public dataset, small test set size, limited patient cohort coverage, use of diagnostic accuracy measures and cross-validation, lack of model external validation and model performance comparison with clinicians, and risk of bias of reference standard are found in the included studies. Hence, their AI-based CAD systems might be unfit for the real clinical situations due to a lack of generalization ability. In the future, more AI-based CAD systems specifically designed/fine-tuned for a wider range of imaging modalities and pathologies in the pediatric radiology should be developed. In addition, more robust study designs should be used in further research to address the aforementioned methodological issues for providing the trustworthy findings to convince the clinical centers to adopt the AI-based CAD in the pediatric radiology. In this way, the potential benefits of the CAD could be realized in a wider context.

## Data Availability

Not applicable.

## References

[1] Lu Y., Zheng N., Ye M., Zhu Y., Zhang G., Nazemi E., He J. (2023). Proposing intelligent approach to predicting air kerma within radiation beams of medical x-ray imaging systems. Diagnostics.

[2] Sun Z., Ng C.K.C. (2022). Artificial intelligence (enhanced super-resolution generative adversarial network) for calcium deblooming in coronary computed tomography angiography: A feasibility study. Diagnostics.

[3] Sun Z., Ng C.K.C. (2022). Finetuned super-resolution generative adversarial network (artificial intelligence) model for calcium deblooming in coronary computed tomography angiography. J. Pers. Med..

[4] Ng C.K.C., Leung V.W.S., Hung R.H.M. (2022). Clinical evaluation of deep learning and atlas-based auto-contouring for head and neck radiation therapy. Appl. Sci..

[5] Choy G., Khalilzadeh O., Michalski M., Do S., Samir A.E., Pianykh O.S., Geis J.R., Pandharipande P.V., Brink J.A., Dreyer K.J. (2018). Current applications and future impact of machine learning in radiology. Radiology.

[6] Chan H.P., Hadjiiski L.M., Samala R.K. (2020). Computer-aided diagnosis in the era of deep learning. Med. Phys..

[7] Suzuki K. (2012). A review of computer-aided diagnosis in thoracic and colonic imaging. Quant. Imaging Med. Surg..

[8] Wardlaw J.M., Mair G., von Kummer R., Williams M.C., Li W., Storkey A.J., Trucco E., Liebeskind D.S., Farrall A., Bath P.M. (2022). Accuracy of automated computer-aided diagnosis for stroke imaging: A critical evaluation of current evidence. Stroke.

[9] Harris M., Qi A., Jeagal L., Torabi N., Menzies D., Korobitsyn A., Pai M., Nathavitharana R.R., Ahmad Khan F. (2019). A systematic review of the diagnostic accuracy of artificial intelligence-based computer programs to analyze chest x-rays for pulmonary tuberculosis. PLoS ONE.

[10] Liu X., Faes L., Kale A.U., Wagner S.K., Fu D.J., Bruynseels A., Mahendiran T., Moraes G., Shamdas M., Kern C. (2019). A comparison of deep learning performance against health-care professionals in detecting diseases from medical imaging: A systematic review and meta-analysis. Lancet Digit. Heal..

[11] Masud R., Al-Rei M., Lokker C. (2019). Computer-aided detection for breast cancer screening in clinical settings: Scoping review. JMIR Med. Inform..

[12] Aggarwal R., Sounderajah V., Martin G., Ting D.S.W., Karthikesalingam A., King D., Ashrafian H., Darzi A. (2021). Diagnostic accuracy of deep learning in medical imaging: A systematic review and meta-analysis. NPJ Digit. Med..

[13] Vasey B., Ursprung S., Beddoe B., Taylor E.H., Marlow N., Bilbro N., Watkinson P., McCulloch P. (2021). Association of clinician diagnostic performance with machine learning-based decision support systems: A systematic review. JAMA Netw. Open.

[14] Ng C.K.C. (2022). Artificial intelligence for radiation dose optimization in pediatric radiology: A systematic review. Children.

[15] Al Mahrooqi K.M.S., Ng C.K.C., Sun Z. (2015). Pediatric computed tomography dose optimization strategies: A literature review. J. Med. Imaging Radiat. Sci..

[16] Schalekamp S., Klein W.M., van Leeuwen K.G. (2022). Current and emerging artificial intelligence applications in chest imaging: A pediatric perspective. Pediatr. Radiol..

[17] Davendralingam N., Sebire N.J., Arthurs O.J., Shelmerdine S.C. (2021). Artificial intelligence in paediatric radiology: Future opportunities. Br. J. Radiol..

[18] Kim K.W., Lee J., Choi S.H., Huh J., Park S.H. (2015). Systematic review and meta-analysis of studies evaluating diagnostic test accuracy: A practical review for clinical researchers-Part I. general guidance and tips. Korean J. Radiol..

[19] Zhao W.J., Fu L.R., Huang Z.M., Zhu J.Q., Ma B.Y. (2019). Effectiveness evaluation of computer-aided diagnosis system for the diagnosis of thyroid nodules on ultrasound: A systematic review and meta-analysis. Medicine.

[20] Devnath L., Summons P., Luo S., Wang D., Shaukat K., Hameed I.A., Aljuaid H. (2022). Computer-aided diagnosis of coal workers’ pneumoconiosis in chest x-ray radiographs using machine learning: A systematic literature review. Int. J. Environ. Res. Public Health.

[21] Groen A.M., Kraan R., Amirkhan S.F., Daams J.G., Maas M. (2022). A systematic review on the use of explainability in deep learning systems for computer aided diagnosis in radiology: Limited use of explainable AI?. Eur. J. Radiol..

[22] Henriksen E.L., Carlsen J.F., Vejborg I.M., Nielsen M.B., Lauridsen C.A. (2019). The efficacy of using computer-aided detection (CAD) for detection of breast cancer in mammography screening: A systematic review. Acta Radiol..

[23] Gundry M., Knapp K., Meertens R., Meakin J.R. (2018). Computer-aided detection in musculoskeletal projection radiography: A systematic review. Radiography.

[24] Waffenschmidt S., Knelangen M., Sieben W., Bühn S., Pieper D. (2019). Single screening versus conventional double screening for study selection in systematic reviews: A methodological systematic review. BMC Med. Res. Methodol..

[25] Ng C.K.C. (2022). A review of the impact of the COVID-19 pandemic on pre-registration medical radiation science education. Radiography.

[26] PRISMA: Transparent Reporting of Systematic Reviews and Meta-Analyses. https://www.prisma-statement.org.

[27] Xu L., Gao J., Wang Q., Yin J., Yu P., Bai B., Pei R., Chen D., Yang G., Wang S. (2020). Computer-aided diagnosis systems in diagnosing malignant thyroid nodules on ultrasonography: A systematic review and meta-analysis. Eur. Thyroid J..

[28] Imrey P.B. (2020). Limitations of meta-analyses of studies with high heterogeneity. JAMA Netw. Open.

[29] Whiting P.F., Rutjes A.W., Westwood M.E., Mallett S., Deeks J.J., Reitsma J.B., Leeflang M.M., Sterne J.A., Bossuyt P.M., QUADAS-2 Group (2011). QUADAS-2: A revised tool for the quality assessment of diagnostic accuracy studies. Ann. Intern. Med..

[30] Dou R., Gao W., Meng Q., Zhang X., Cao W., Kuang L., Niu J., Guo Y., Cui D., Jiao Q. (2022). Machine learning algorithm performance evaluation in structural magnetic resonance imaging-based classification of pediatric bipolar disorders type I patients. Front. Comput. Neurosci..

[31] Kuttala D., Mahapatra D., Subramanian R., Oruganti V.R.M. (2022). Dense attentive GAN-based one-class model for detection of autism and ADHD. J. King Saud Univ. Comput. Inf. Sci..

[32] Li M., Wang H., Shang Z., Yang Z., Zhang Y., Wan H. (2020). Ependymoma and pilocytic astrocytoma: Differentiation using radiomics approach based on machine learning. J. Clin. Neurosci..

[33] Peruzzo D., Arrigoni F., Triulzi F., Righini A., Parazzini C., Castellani U. (2016). A framework for the automatic detection and characterization of brain malformations: Validation on the corpus callosum. Med. Image Anal..

[34] Prince E.W., Whelan R., Mirsky D.M., Stence N., Staulcup S., Klimo P., Anderson R.C.E., Niazi T.N., Grant G., Souweidane M. (2020). Robust deep learning classification of adamantinomatous craniopharyngioma from limited preoperative radiographic images. Sci. Rep..

[35] Tan L., Chen Y., Maloney T.C., Caré M.M., Holland S.K., Lu L.J. (2013). Combined analysis of sMRI and fMRI imaging data provides accurate disease markers for hearing impairment. Neuroimage Clin..

[36] Xiao Z., Wu J., Wang C., Jia N., Yang X. (2019). Computer-aided diagnosis of school-aged children with ASD using full frequency bands and enhanced SAE: A multi-institution study. Exp. Ther. Med..

[37] Zahia S., Garcia-Zapirain B., Saralegui I., Fernandez-Ruanova B. (2020). Dyslexia detection using 3D convolutional neural networks and functional magnetic resonance imaging. Comput. Methods Programs Biomed..

[38] Zhou X., Lin Q., Gui Y., Wang Z., Liu M., Lu H. (2021). Multimodal MR images-based diagnosis of early adolescent attention-deficit/hyperactivity disorder using multiple kernel learning. Front. Neurosci..

[39] Lee H., Eun Y., Hwang J.Y., Eun L.Y. (2022). Explainable deep learning algorithm for distinguishing incomplete Kawasaki disease by coronary artery lesions on echocardiographic imaging. Comput. Methods Programs Biomed..

[40] Petibon Y., Fahey F., Cao X., Levin Z., Sexton-Stallone B., Falone A., Zukotynski K., Kwatra N., Lim R., Bar-Sever Z. (2021). Detecting lumbar lesions in 99m Tc-MDP SPECT by deep learning: Comparison with physicians. Med. Phys..

[41] Sezer A., Sezer H.B. (2020). Deep convolutional neural network-based automatic classification of neonatal hip ultrasound images: A novel data augmentation approach with speckle noise reduction. Ultrasound Med. Biol..

[42] Behzadi-Khormouji H., Rostami H., Salehi S., Derakhshande-Rishehri T., Masoumi M., Salemi S., Keshavarz A., Gholamrezanezhad A., Assadi M., Batouli A. (2020). Deep learning, reusable and problem-based architectures for detection of consolidation on chest x-ray images. Comput. Methods Programs Biomed..

[43] Bodapati J.D., Rohith V.N. (2022). ChxCapsNet: Deep capsule network with transfer learning for evaluating pneumonia in paediatric chest radiographs. Measurement.

[44] Helm E.J., Silva C.T., Roberts H.C., Manson D., Seed M.T., Amaral J.G., Babyn P.S. (2009). Computer-aided detection for the identification of pulmonary nodules in pediatric oncology patients: Initial experience. Pediatr. Radiol..

[45] Jiang Z., Chen L. (2022). Multisemantic level patch merger vision transformer for diagnosis of pneumonia. Comput. Math. Method Med..

[46] Liang G., Zheng L. (2020). A transfer learning method with deep residual network for pediatric pneumonia diagnosis. Comput. Methods Programs Biomed..

[47] Mahomed N., van Ginneken B., Philipsen R.H.H.M., Melendez J., Moore D.P., Moodley H., Sewchuran T., Mathew D., Madhi S.A. (2020). Computer-aided diagnosis for World Health Organization-defined chest radiograph primary-endpoint pneumonia in children. Pediatr. Radiol..

[48] Shouman M.A., El-Fiky A., Hamada S., El-Sayed A., Karar M.E. (2022). Computer-assisted lung diseases detection from pediatric chest radiography using long short-term memory networks. Comput. Electr. Eng..

[49] Silva L., Araújo L., Ferreira V., Neto R., Santos A. (2022). Convolutional neural networks applied in the detection of pneumonia by x-ray images. Int. J. Innov. Comput. Appl..

[50] Vrbančič G., Podgorelec V. (2022). Efficient ensemble for image-based identification of pneumonia utilizing deep CNN and SGD with warm restarts. Expert Syst. Appl..

[51] Guan Y., Peng H., Li J., Wang Q. (2022). A mutual promotion encoder-decoder method for ultrasonic hydronephrosis diagnosis. Methods.

[52] Zheng Q., Furth S.L., Tasian G.E., Fan Y. (2019). Computer-aided diagnosis of congenital abnormalities of the kidney and urinary tract in children based on ultrasound imaging data by integrating texture image features and deep transfer learning image features. J. Pediatr. Urol..

[53] Kleinfelder T.R., Ng C.K.C. (2022). Effects of image postprocessing in digital radiography to detect wooden, soft tissue foreign bodies. Radiol. Technol..

[54] Sun Z., Ng C.K.C., Wong Y.H., Yeong C.H. (2021). 3D-printed coronary plaques to simulate high calcification in the coronary arteries for investigation of blooming artifacts. Biomolecules.

[55] Ng C.K.C., Sun Z. (2010). Development of an online automatic computed radiography dose data mining program: A preliminary study. Comput. Methods Programs Biomed..

[56] Christe A., Peters A.A., Drakopoulos D., Heverhagen J.T., Geiser T., Stathopoulou T., Christodoulidis S., Anthimopoulos M., Mougiakakou S.G., Ebner L. (2019). Computer-aided diagnosis of pulmonary fibrosis using deep learning and CT images. Investig. Radiol..

[57] Tanaka H., Chiu S.W., Watanabe T., Kaoku S., Yamaguchi T. (2019). Computer-aided diagnosis system for breast ultrasound images using deep learning. Phys. Med. Biol..

[58] Kim H.L., Ha E.J., Han M. (2019). Real-world performance of computer-aided diagnosis system for thyroid nodules using ultrasonography. Ultrasound Med. Biol..

[59] Jeongy E.Y., Kim H.L., Ha E.J., Park S.Y., Cho Y.J., Han M. (2019). Computer-aided diagnosis system for thyroid nodules on ultrasonography: Diagnostic performance and reproducibility based on the experience level of operators. Eur. Radiol..

[60] Park H.J., Kim S.M., La Yun B., Jang M., Kim B., Jang J.Y., Lee J.Y., Lee S.H. (2019). A computer-aided diagnosis system using artificial intelligence for the diagnosis and characterization of breast masses on ultrasound: Added value for the inexperienced breast radiologist. Medicine.

[61] Zhang S., Sun F., Wang N., Zhang C., Yu Q., Zhang M., Babyn P., Zhong H. (2019). Computer-aided diagnosis (CAD) of pulmonary nodule of thoracic CT image using transfer learning. J. Digit. Imaging.

[62] Wei R., Lin K., Yan W., Guo Y., Wang Y., Li J., Zhu J. (2019). Computer-aided diagnosis of pancreas serous cystic neoplasms: A radiomics method on preoperative MDCT images. Technol. Cancer Res. Treat..

[63] Chen C.H., Lee Y.W., Huang Y.S., Lan W.R., Chang R.F., Tu C.Y., Chen C.Y., Liao W.C. (2019). Computer-aided diagnosis of endobronchial ultrasound images using convolutional neural network. Comput. Methods Programs Biomed..

[64] Gong J., Liu J., Hao W., Nie S., Wang S., Peng W. (2019). Computer-aided diagnosis of ground-glass opacity pulmonary nodules using radiomic features analysis. Phys. Med. Biol..

[65] Li L., Liu Z., Huang H., Lin M., Luo D. (2019). Evaluating the performance of a deep learning-based computer-aided diagnosis (DL-CAD) system for detecting and characterizing lung nodules: Comparison with the performance of double reading by radiologists. Thorac. Cancer..

[66] Bajaj V., Pawar M., Meena V.K., Kumar M., Sengur A., Guo Y. (2019). Computer-aided diagnosis of breast cancer using bi-dimensional empirical mode decomposition. Neural Comput. Appl..

[67] Nayak A., Baidya Kayal E., Arya M., Culli J., Krishan S., Agarwal S., Mehndiratta A. (2019). Computer-aided diagnosis of cirrhosis and hepatocellular carcinoma using multi-phase abdomen CT. Int. J. Comput. Assist. Radiol. Surg..

[68] Greer M.D., Lay N., Shih J.H., Barrett T., Bittencourt L.K., Borofsky S., Kabakus I., Law Y.M., Marko J., Shebel H. (2018). Computer-aided diagnosis prior to conventional interpretation of prostate mpMRI: An international multi-reader study. Eur. Radiol..

[69] Ishioka J., Matsuoka Y., Uehara S., Yasuda Y., Kijima T., Yoshida S., Yokoyama M., Saito K., Kihara K., Numao N. (2018). Computer-aided diagnosis of prostate cancer on magnetic resonance imaging using a convolutional neural network algorithm. BJU Int..

[70] Song Y., Zhang Y.D., Yan X., Liu H., Zhou M., Hu B., Yang G. (2018). Computer-aided diagnosis of prostate cancer using a deep convolutional neural network from multiparametric MRI. J. Magn. Reason. Imaging..

[71] Yoo Y.J., Ha E.J., Cho Y.J., Kim H.L., Han M., Kang S.Y. (2018). Computer-aided diagnosis of thyroid nodules via ultrasonography: Initial clinical experience. Korean J. Radiol..

[72] Al-Antari M.A., Al-Masni M.A., Choi M.T., Han S.M., Kim T.S. (2018). A fully integrated computer-aided diagnosis system for digital x-ray mammograms via deep learning detection, segmentation, and classification. Int. J. Med. Inform..

[73] Choi Y.J., Baek J.H., Park H.S., Shim W.H., Kim T.Y., Shong Y.K., Lee J.H. (2017). A computer-aided diagnosis system using artificial intelligence for the diagnosis and characterization of thyroid nodules on ultrasound: Initial clinical assessment. Thyroid.

[74] Li S., Jiang H., Wang Z., Zhang G., Yao Y.D. (2018). An effective computer aided diagnosis model for pancreas cancer on PET/CT images. Comput. Methods Programs Biomed..

[75] Chang C.C., Chen H.H., Chang Y.C., Yang M.Y., Lo C.M., Ko W.C., Lee Y.F., Liu K.L., Chang R.F. (2017). Computer-aided diagnosis of liver tumors on computed tomography images. Comput. Methods Programs Biomed..

[76] Cho E., Kim E.K., Song M.K., Yoon J.H. (2018). Application of computer-aided diagnosis on breast ultrasonography: Evaluation of diagnostic performances and agreement of radiologists according to different levels of experience. J. Ultrasound Med..

[77] Sun Z., Ng C.K.C., Sá Dos Reis C. (2018). Synchrotron radiation computed tomography versus conventional computed tomography for assessment of four types of stent grafts used for endovascular treatment of thoracic and abdominal aortic aneurysms. Quant. Imaging Med. Surg..

[78] Almutairi A.M., Sun Z., Ng C., Al-Safran Z.A., Al-Mulla A.A., Al-Jamaan A.I. (2010). Optimal scanning protocols of 64-slice CT angiography in coronary artery stents: An in vitro phantom study. Eur. J. Radiol..

[79] Sun Z., Ng C.K.C. (2017). Use of synchrotron radiation to accurately assess cross-sectional area reduction of the aortic branch ostia caused by suprarenal stent wires. J. Endovasc. Ther..

[80] Zebari D.A., Ibrahim D.A., Zeebaree D.Q., Haron H., Salih M.S., Damaševičius R., Mohammed M.A. (2021). Systematic review of computing approaches for breast cancer detection based computer aided diagnosis using mammogram images. Appl. Artif. Intell..

